# NLRP1 Inflammasomes: A Potential Target for the Treatment of Several Types of Brain Injury

**DOI:** 10.3389/fimmu.2022.863774

**Published:** 2022-05-30

**Authors:** Liang Mi, Xiaobin Min, Yan Chai, Jianning Zhang, Xin Chen

**Affiliations:** ^1^ Department of Neurosurgery, Tianjin Medical University General Hospital, Tianjin, China; ^2^ Tianjin Neurological Institute, Key Laboratory of Posttrauma Neurorepair and Regeneration in Central Nervous System, Ministry of Education, Tianjin Key Laboratory of Injuries, Variations and Regeneration of Nervous System, Tianjin, China; ^3^ Baodi Clinical College, Tianjin Medical University, Tianjin, China

**Keywords:** NLRP1 inflammasome, subarachnoid hemorrhage, stroke, traumatic brain injury, neuroinflammation

## Abstract

NOD-like receptor (NLR) family pyrin domain-containing 1 (NLRP1) is a member of the NLR family. The NLRP1 inflammasome consists of the NLRP1 protein, the adaptor protein apoptosis-associated speck-like protein containing a CARD domain, and the effector molecule pro-caspase-1. When stimulated, the inflammasome initiates the cleavage of pro-caspase-1 and converts it into its active form, caspase-1; then, caspase-1 facilitates the cleavage of the proinflammatory cytokines interleukin-1β and interleukin-18 into their active and secreted forms. In addition, caspase-1 also mediates the cleavage of gasdermin D, which leads to pyroptosis, an inflammatory form of cell death. Pathological events that damage the brain and result in neuropathological conditions can generally be described as brain injury. Neuroinflammation, especially that driven by NLRP1, plays a considerable role in the pathophysiology of brain injury, such as early brain injury (EBI) of subarachnoid hemorrhage, ischemic brain injury during stroke, and traumatic brain injury (TBI). In this article, a thorough overview of NLRP1 is presented, including its structure, mechanism of activation, and role in neuroinflammation. We also present recent studies on NLRP1 as a target for the treatment of EBI, ischemic brain injury, TBI, and other types of brain injury, thus highlighting the perspective of NLRP1 as an effective mediator of catastrophic brain injury.

## Introduction

Subarachnoid hemorrhage (SAH) is commonly initiated by aneurysm rupture and leads to poor neurological outcomes, along with high morbidity and mortality (1, 2). As a subtype of stroke, SAH is characterized by two core factors that are blamed for its unfavorable consequences: early brain injury (EBI) and cerebral vasospasm (CVS). Emerging evidence has shown that EBI plays a more significant role than CVS in the outcomes of SAH (3–5). The toxicity of subarachnoid blood and the temporary global ischemia induce an extreme immune reaction during the EBI stage, which is responsible for secondary brain injury (6).

The potential mechanisms of brain injury after SAH include endoplasmic reticulum stress (ERS), oxidative stress, neuroinflammation, apoptosis, and autophagy (7–9). Many studies have explored the relationship between NOD-like receptor (NLR) family pyrin domain-containing 3 (NLRP3) and SAH, thus indicating that NLRP3 is a potential mediator of SAH therapy. Extreme ERS can cause oxidative stress and induce downstream responses, resulting in inflammation (10). The stimulation of thioredoxin-interacting protein/NLRP3 may be related to the inflammatory response in brain injury *via* ERS (6). In addition, NLRP3 regulates neuronal pyroptosis in EBI following SAH (11). Currently, the role of NLRP1 in EBI is becoming increasingly attractive. The level of NLRP1 increases during the EBI period (12), and NLRP1 is to decreased in the atorvastatin-treated SAH mouse model (13). Thus, it is reasonable to consider NLRP1 a promising target for the treatment of SAH.

Stroke occurs when the blood flow to the brain declines abruptly, which often leads to neurological functional impairment or deficiency (14). As the most common type of stroke, ischemic stroke accounts for approximately 80% of all stroke incidents (15) and can be subdivided into two types: global ischemia and focal ischemia (16, 17). The pathology of ischemic brain injury consists of a central ischemic core and the surrounding peri-infarct zone caused by focal hypoperfusion (18).

Neuroinflammation plays a pivotal role in poststroke physiopathology and results in an imbalance in tissue homeostasis (19, 20), such as reactive oxygen species (ROS), imbalanced cytoplasmic Ca^2+^, impairment of the blood–brain barrier (BBB), and mitochondrial stress (21–23). NLRP1 can promote the production of proinflammatory cytokines, thus causing the death of neuronal cells and behavioral dysfunction (24). However, the maturation of interleukin-1β (IL-1β) and interleukin-18 (IL-18) in mice with focal cerebral stroke can be limited by restraining the NLRP1 inflammasome (25). These results demonstrate that the NLRP1 inflammasome may play an important role in ischemic brain injury and may be a promising target for the treatment of ischemic stroke.

Traumatic brain injury (TBI) is a subtype of brain injury caused by contact sports, traffic accidents, or warfare and often leads to morbidity and mortality among children and young adults (26). Generally, after a mechanical insult to the brain, TBI results in a primary injury that initiates a secondary cascade of events. The primary injury directly leads to the loss of neurons and necrotic death (27), while the secondary brain insult is characterized by a series of neuroinflammatory responses, including oxidative stress, mitochondrial dysfunction, BBB leakage, activation of microglia and astrocytes, and an increase in cytokines (28, 29).

Several inflammasomes are closely associated with TBI, such as the NLRP3 and AIM2 inflammasomes (30–32). In recent decades, emerging clinical evidence has demonstrated that NLRP1 is an ideal target inflammasome for TBI therapy—for example, TBI patients with clinically diagnosed unfavorable outcomes possessed higher levels of NLRP1 expression in their cerebrospinal fluid (CSF) than those with favorable outcomes (33). In addition, CSF collected from a spinal cord injury and TBI patients was found to contain exosomes carrying NLRP1 (34).

## The Innate Immune System and Inflammation in the Central Nervous System

The innate immune system protects the host from infections and noninfectious harmful stimuli, and its key role is to detect and react to pathogens. In some cases, immune cells directly identify microbial ligands (such as bacterial flagellin and double-stranded RNA) or detect distinct pathogen motifs known as pathogen-associated molecular patterns (35). In other cases, innate immune receptors recognize molecules related to a sterile tissue damage called danger-associated molecular patterns (36). Germline-encoded pattern recognition receptors (PRRs), such as Toll-like receptors, C-type lectin receptors (CLRs), and nucleotide oligomerization domain-like receptors (NOD-like receptors or NLRs), can identify these signals (37). After being engaged, these receptors facilitate the maturation and secretion of proinflammatory cytokines and molecules, leading to inflammation and the removal of pathogens.

Because of its primary resident immune cells, which are microglia and astrocytes, the CNS is protected from external damage, pathogens, and toxins (38, 39). Equally important is the integrity of the CNS which is also sustained by the extremely selective semipermeable membrane barrier, known as the BBB, which separates the CNS from the periphery (40). In response to infections, resident immune cells are able to produce innate immune responses and stimulate inflammation in the CNS, which is often referred to as neuroinflammation. Interestingly, it has been demonstrated that neurons are also involved in this kind of response by expressing PRRs and producing proinflammatory cytokines (41, 42). While inflammation protects the host from infections, excessive inflammation can cause severe tissue injury and even death by amplifying harmful pathways (43).

## The NLRP1-ASC-Caspase-1 Inflammasome Complex and Its Role in Neuroinflammation

**Figure 1:**
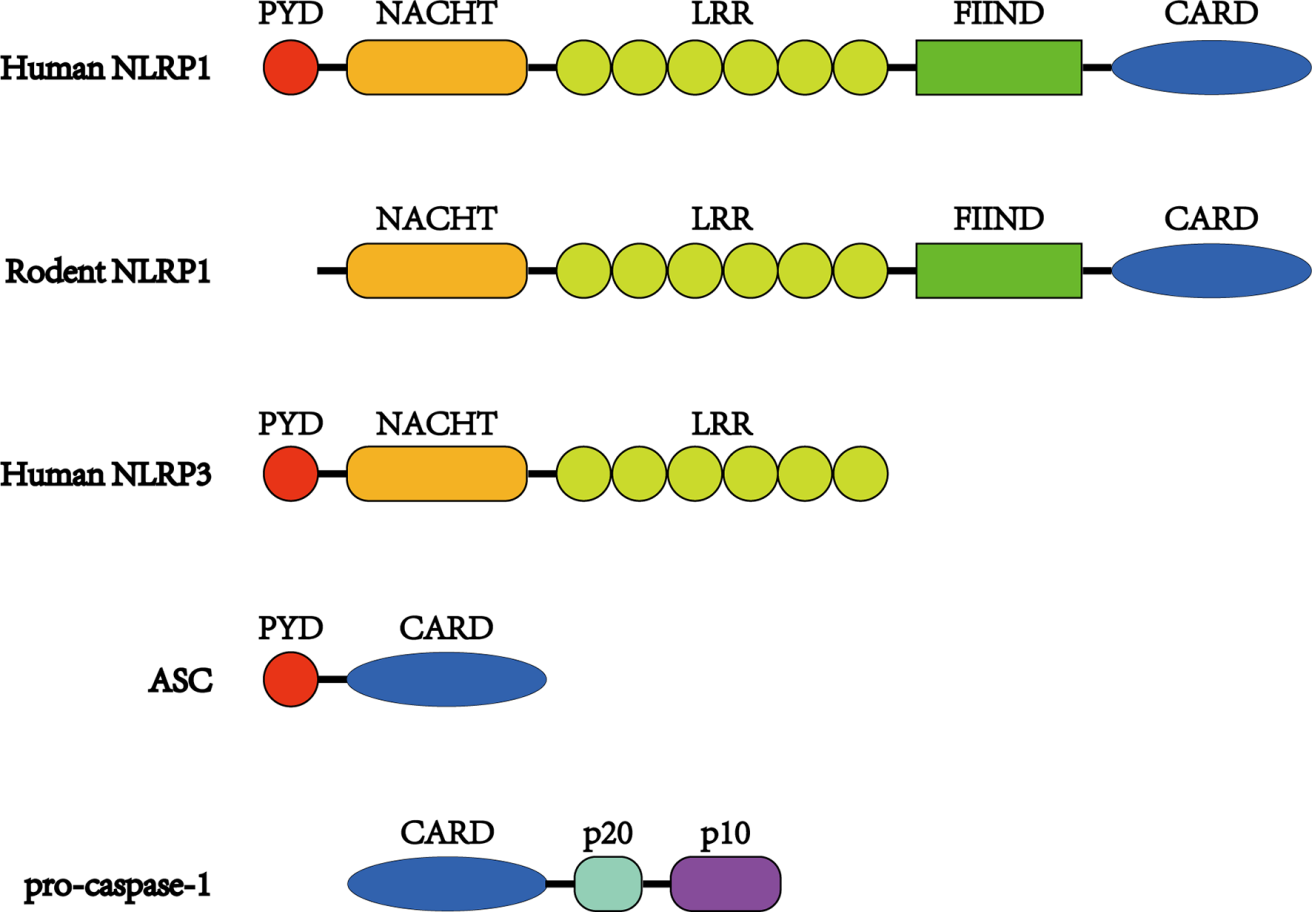
Structures of NLRP1 inflammasome proteins. Unlike other NLRs, NLRP1 proteins have a FIIND and CARD at the C-terminus. The FIIND consists of ZU5 and UPA subdomains, and autoproteolysis takes place between them. In addition, NLRP1 proteins contain NACHT and LRR domains preceding the FIIND. Human NLRP1 also possesses a PYD domain at the N-terminus, which is absent in its rodent orthologs. ASC consists of a PYD and CARD, while pro-caspase-1 possesses a CARD before its catalytic p20 and p10 subunits.

**Figure 2:**
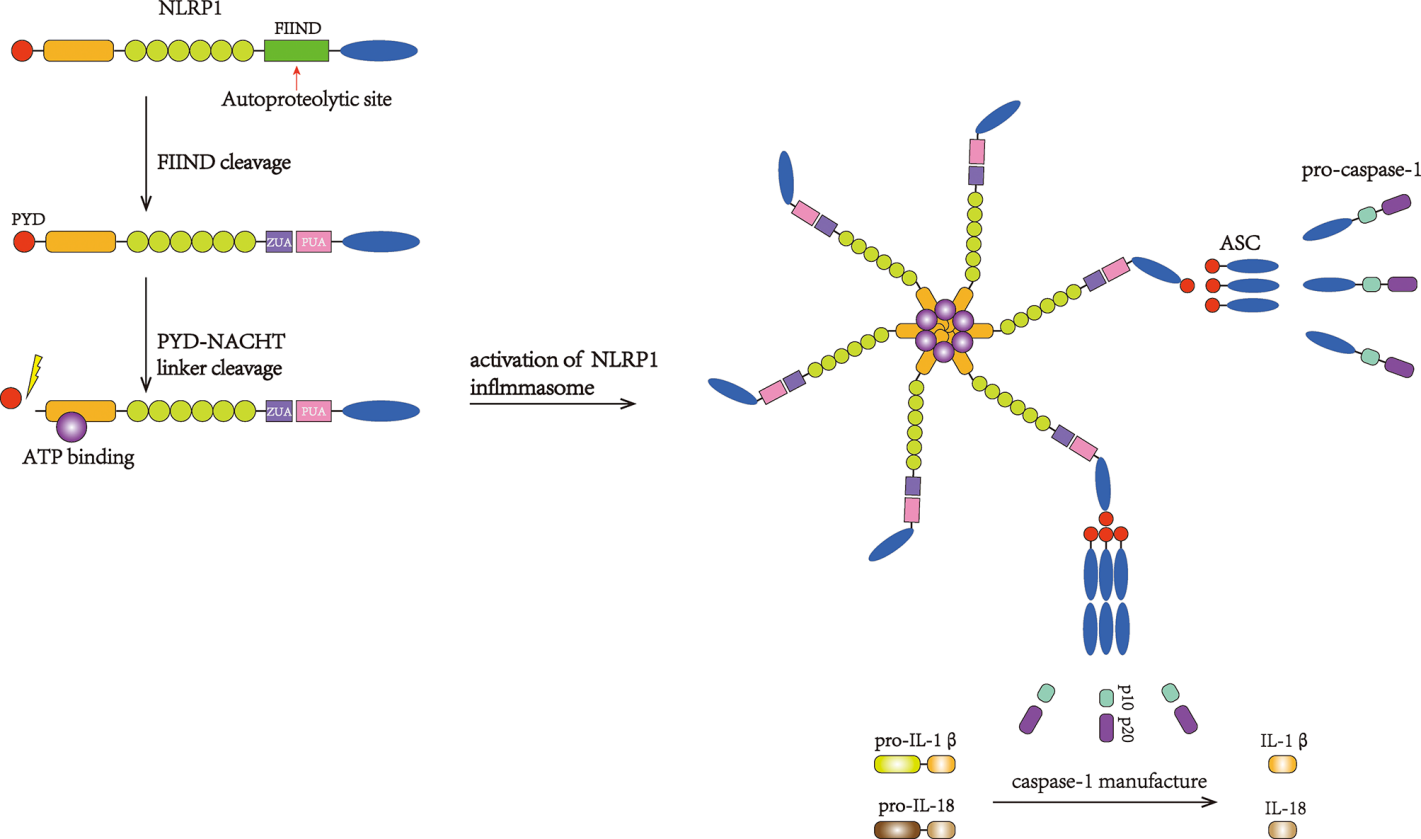
Activation of the NLRP1 inflammasome. FIIND is autocleaved to activate NLRP1. Then, PYD undergoes cleavage, and the inflammasome complex subsequently forms, suggesting that PYD may play an autoinhibitory role. The binding of ASC can recruit pro-caspase-1. Then, pro-caspase-1 undergoes autocatalytic cleavage and forms the p10 and p20 subunits, which initiate the maturation and secretion of IL-1β and IL-18.

NLRP1 was the first inflammasome to have been studied in length and is also known as NALP1, NAC, DEFCAP, CLR17.1, and CARD7 (44). NLRP1 is expressed mainly in motor neurons in the cerebral cortex and spinal cord and is also present in microglia (45). As a multiprotein complex, the NLRP1 inflammasome is composed of NLRP1, the adaptor protein apoptosis-associated speck-like protein containing a CARD domain (ASC) and the effector protein pro-caspase-1 (46). ASC possesses both a pyrin domain (PYD) and a CARD domain and links NLRP1 and pro-caspase-1 (**Figure 1**). After being stimulated, NLRP1 assembles ACS through homotypic interactions between the PYDs; subsequently, prion-like polymerization of ASC is catalyzed by NLRP1 to facilitate a downstream reaction (47–49). As a member of the cysteine-aspartic acid protease family, pro-caspase-1 then attaches to the complex through CARD–CARD interactions, favoring ASC oligomerization. Many pro-caspase-1 molecules are recruited and undergo autocatalytic cleavage to produce p10 and p20 subunits. These subunits transform into two heterodimers that are the activated forms of caspase-1 (46, 50). Once stimulated, caspase-1 initiates the cleavage of IL-1β and IL-18 into their mature forms and mediates their secretion (25). IL-1β and IL-18 are proinflammatory cytokines that can activate innate and adaptive immune responses, such as inflammation (51) (**Figure 2**).

The NLRP1 inflammasome is the first member of the NLR family and is highly expressed in the brain (52). The stimulation of NLRP1 can result in an inflammatory response (53); in several animal models, it has been discovered that inflammatory reactions and brain injury can be moderated by restraining the NLRP1 inflammasome (54–56). Active caspase-1 could cleave the pore-forming protein gasdermin D and cause pyroptosis, a type of programmed proinflammatory neuronal death that differs from apoptosis (46). Pyroptosis consists of osmotic swelling, the formation of pores in the membrane, and the disruption of membrane integrity (57); this process promotes the release of the inflammasome into the extracellular space and induces brain inflammation (58).

## The Structure of the NLRP1 Protein

As a member of the NLR family, human NLRP1 (hNLRP1) is characterized by 1,473 amino acids and 165.9 kDa and contains a primary NACHT (NAIP, CIITA, HET-E, and TP-1) domain (also known as nucleotide oligomerization domain) and a leucine-rich repeat (LRR) domain, along with a PYD located at its N-terminus (59, 60). Unlike other NLRs, hNLRP1 has a C-terminal extension containing a function-to-find domain (FIIND) flanked by an LRR and a CARD attached to the C-terminus. Structurally, the FIIND contains a combination of ZU5 and UPA subdomains and undergoes posttranslational proteolytic cleavage (**Figure 1**). The cleavage, which is essential for the activation of NLRP1, leads to the separation of the ZU5 and UPA domains, which remain associated with each other through a noncovalent linkage (61–63). It has also been shown that only a portion of NLRP1 proteins undergo autoproteolysis (~50%) (62).

PYD and CARD domains are members of the death domain superfamily, which is known for the transduction of apoptosis and inflammatory signals (64, 65). CARD has been proven to be the necessary effector domain, while PYD seems to be less important, as it is the C-terminal CARD and not the N-terminal PYD that assembles ASC to form the inflammasome (62). Although several CARD domains can recruit pro-caspase-1 in the absence of ASC (66, 67), ASC is essential for bridging hNLRP1 CARD and pro-caspase-1.

In addition to the cleavage of the FIIND, hNLRP1 also undergoes N-terminal cleavage at the linker sequence between PYD and NACHT. The proteolytic cleavage of the N-terminus has been shown to be a common molecular mechanism for NLRP1 activation (68, 69). In 2016, Chavarría-Smith *et al.* performed artificial cleavage at the linker region, and the reformed hNLRP1 was activated (69). Interestingly, even when the PYD was substituted with a green fluorescent protein, this activation occurred. N-terminal cleavage is also necessary in mouse Nlrp1b, which lacks a PYD (68). Thus, there are reasons to believe that the N-terminus acts as an autoinhibitor instead of an inflammasome signal transductor. However, further investigation is required to elucidate the role of PYD in the activation of NLRP1.

Unlike NLRP1, which is the only gene in humans, the mouse genome contains three paralogues (Nlrp1a, Nlrp1b, and Nlrp1c), although it is predicted that Nlrp1c is a pseudogene (70, 71). In the absence of a PYD, both mouse NLRP1A (mNLRP1A) and mouse NLRP1B (mNLRP1b) can assemble pro-caspase-1 regardless of the presence of ASC (53, 66, 72). mNLRP1B possesses at least five comparatively polymorphic alleles (70). In a study on lethal toxin-induced macrophages, the researchers observed that allele 1 was carried by the most susceptible macrophage strains and that limited numbers of susceptible macrophage strains carry allele 5, whereas all resistant macrophages carried alleles 2, 3, or 4 (70). Because of deficient autoproteolysis and truncation prior to the CARD domain, both mNLRP1B alleles 3 and 4 lack functional abilities (63, 70, 73). Similar to mNLRP1, rat NLRP1 (rNLRP1) does not have a PYD at the N-terminus but consists of only one Nlrp1 gene (**Figure 1**). There are at least five rNLRP1 alleles, all of which encode functional proteins (73, 74). However, the role of ASC in the recruitment of the rNLRP1 inflammasome remains unknown.

It has been demonstrated that NLRP1 exhibits structural and functional differences between humans and rodent, which result in difficulties in investigating NLRP1 (75). In view of the limited knowledge regarding the first inflammasome described, more efforts are needed to study the mechanisms of NLRP1.

## Activators That Stimulate NLRP1

In recent years, several stimuli have been shown to be associated with NLRP1 activation, and these stimuli can be divided into two groups. The first group consists of direct activators, such as the anthrax lethal toxin (LT) and *Shigella flexneri*, which can activate only one NLRP1 allele subset *via* the direct modification and degradation of the NLRP1 N-terminal fragment (69, 76, 77). The other group of indirect activators consists of *Toxoplasma gondii* (T pathogen), inhibitor of dipeptidyl peptidases 8 and 9 (DPP8/DPP9), and metabolic inhibitors (73, 78–81) and seems to cause a type of cellular disorder that can be detected by NLRP1 proteins.

### Direct Activators

**Figure 3:**
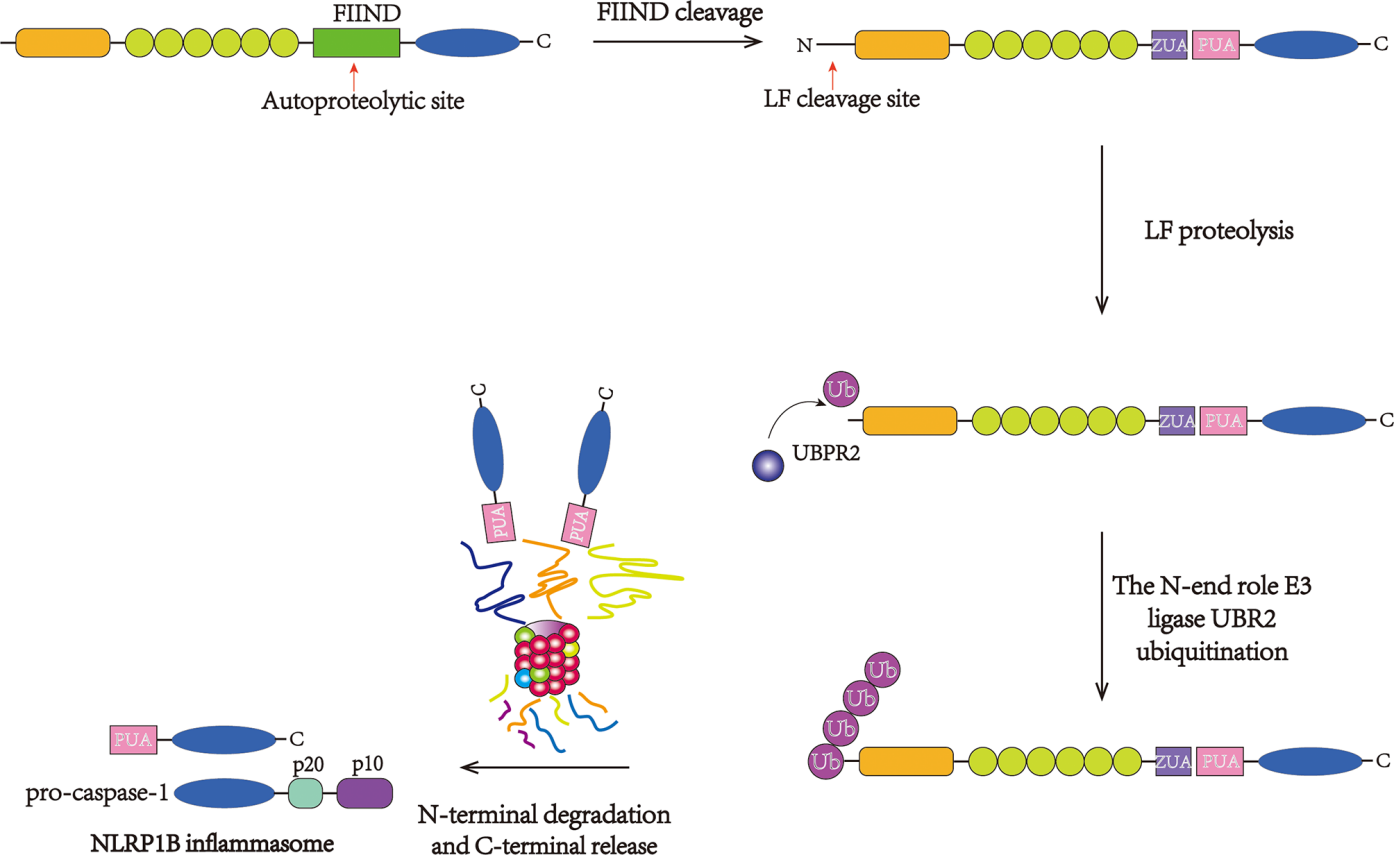
Direct activation of the NLRP1B inflammasome. NLRP1B undergoes autoproteolysis at the FIIND and generates the ZU5 and UPA subdomains. Then, LF cleavage between the K44 and L45 residues in the N-terminus produces an unstable N-terminal residue. The neo-N-terminus is subsequently recognized and ubiquitinated by the N-end rule E3 ligase UBR2, initiating proteasome-mediated degradation. The C-terminal fragment is free to recruit and stimulate pro-caspase-1.

Macrophages with mNlrp1B alleles 1 and 5 or with rNlrp1 alleles 1 and 2 can be activated by LT (82, 83). After the FIIND undergoes posttranslational autoproteolysis, the subdomains ZU5 and UPA produce C-terminal and N-terminal fragments, respectively, which associate with each other *via* noncovalent bonding. In 2019, Chui AJ and Sandstrom A *et al.* showed that an N-terminus was produced after NLRP1 cleavage by LF, which can be identified by the N-end rule pathway (77, 84). The N-end rule E3 ligase UBR2 (85, 86) plays an important role in recognizing and ubiquitinating the neo-N-terminus (84, 87), while the cleavage in the FIIND domain blocks the degradation of the C-terminal fragment during the cleavage of the N-terminal fragment, thus liberating the C-terminal fragment and resulting in the subsequent assembly and activation of pro-caspase-1 and pyroptosis (**Figure 3**). This model explains the demand for proteolytic cleavage and FIIND autoproteolysis during inflammasome activation and demonstrates that LT is a danger-associated activator of rodent NLRP1.

Correspondingly, *S. flexneri*, an intracellular bacterium, was discovered to secrete IpaH7.8 E3 ubiquitin ligase, which is capable of ubiquitinating mNlrp1B allele 1 and subsequently activating it (77). Surprisingly, the stimulation of E3 ligase and the host proteasome is required for this process. However, the N-end rule pathway is dispensable for this process, as IpaH7.8 can ubiquitinate the inflammasome itself.

In addition, in 2020, Robinson KS *et al.* discovered that enteroviral 3C protease could directly cleave hNLRP1 at the site between Glu130 and Gly131, which is mapped to the linker region, followed by the PYD domain, and this process activated the NLRP1 inflammasome and induced the secretion of IL-18, providing a novel mechanism for direct NLRP1 inflammasome activation (88).

In summary, this decoy model hypothesizes that, within innate immune cells, pathogens such as LT and *S. flexneri* survive and destroy host NLR proteins that can inhibit pathogen replication. The effectors also degrade the N-terminus of NLRP1 because of its resemblance to the intended targets and thus induce an immune response (77, 89). In addition to acting as a decoy to detect an overwhelming variety of pathogens that undergo constant evolution, this model also provides an explanation for the high polymorphism of NLRP1. More efforts are needed to validate that NLRP1 serves as a decoy. In particular, the hypothesis that pathogen effectors also destroy the intended targets, such as other NLR proteins, as the model assumes, has not yet been proven.

### Indirect Activators

In 2014, it was demonstrated that, in Lewis rat bone marrow-derived macrophages, when NLRP1 was downregulated by siRNA, cell death caused by *T. gondii* was decreased, and the opposite effect was observed when the Nlrp1 allele 5 was overexpressed in CDF macrophages, indicating that the pyroptosis induced by *T. gondii* was closely related to NLRP1 (78). Thus, *T. gondii* became the second stimulus associated with pathogens after LT. Interestingly, in human MonoMac6 cells, when shRNA was used to knockdown NLRP1, enhanced cell death caused by *T. gondii* was reported (90), which seems contradictory to the role of NLRP1 in rats.

In the last few years, VbP, a nonselective inhibitor with specific activity against the dipeptidyl peptidases DPP8 and DPP9 (91), has been shown to stimulate both mouse and rat NLRP1 inflammasomes (92, 93). In addition, NLRP1-induced pyroptosis was observed in human keratinocytes treated with VbP (94), indicating that VbP can not only activate rodent Nlrp1 but also modify hNLRP1. To stimulate NLRP1, VbP degrades the N-termini of sensitive PRRs by proteolysis, liberating the C-terminal fragments to establish inflammasomes (84, 95). Intriguingly, it seems that VbP does not directly cleave N-terminal fragments (93, 95), which suggests that pyroptosis caused by NLRP1 is independent of the N-end rule pathway. In addition, DPP9 inhibits hNLRP1 activation by associating with the FIIND (94).

Mogridge J *et al.* found that 2-deoxyglucose, an inhibitor of glycolysis, plus sodium azide, an oxidative phosphorylation inhibitor, was able to stimulate the NLRP1B allele 1, particularly in HT1080 cells, and this process was independent of the direct cleavage of the N-terminus (80, 81), similar to the effects of *T. gondii* and DPP8/9 inhibitors. Similarly, NLRP1B activation was observed in RAW 264.7 cells after *Listeria monocytogenes* or *S. flexneri* exposure (96); the authors suggests that all three stimuli activated the cells by reducing adenosine triphosphate (ATP), as decreased cytosolic ATP levels were discovered during these processes.

**Figure 4:**
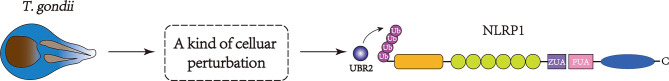
The pathogen-associated effects induced by *T. gondii* infection, DPP8/9 inhibitors, or metabolic inhibitors can be detected by the indirect activation of the NLRP1 inflammasome. These activities can initiate perturbations in cells, which may stimulate the E3 ligase, resulting in the ubiquitination and degradation of the N-terminus of NLRP1.

In contrast to direct stimuli, indirect activators may cause disruptions within cells, stimulating E3 ligase to ubiquitinate the N-terminus of NLRP1 and resulting in its degradation (73). To some degree, in this model, indirect activators sense activities associated with pathogens (80, 81), detecting disturbances in cellular homeostasis and the relationship with metabolism (96–98) (**Figure 4**). However, the detailed mechanism of cell perturbations induced by *T. gondii*, VbP, or metabolic inhibitors remains to be identified, and the specific relationship between these factors has not been reported.

## Activation Mechanism of NLRP1

In hNLRP1, the FIIND contains Ser1213, which can be deprotonated by the highly conserved His1186 residue. The FIIND is structurally divided into the ZU5 and UPA domains. Following posttranslational autoproteolytic cleavage, the two domains remain attached to each other *via* noncovalent bonding (61–63); the autocleavage of FIIND is fundamental for the subsequent activation of NLRP1. Moreover, the NOD domain of hNLRP1 possesses Walker-A and Walker-B, two motifs that bind ATP, which is essential for self-oligomerization and inflammasome recruitment, and NOD plays an important role in forming the core of the inflammasome complex (99). This activation prompts a structural conformational shift that turns NLRP1 into an asteroidal filament-shaped conformation that clusters within NODs. The formation of the inflammasome complex is then achieved through the interaction with ASC and the assembly of pro-caspase-1 (46) (**Figure 2**).

The PYD domain located at the N-terminus interacts with ASC through homotypic PYD–PYD attraction, and pro-caspase-1 is assembled through the interaction of the ASC CARDs and pro-caspase-1. Having its own CARD domain to transduce signals, NLRP1 can recruit caspase-1 independently of ASC. In 2008, a study conducted by Hsu LC revealed that NLRP1 was required to associate with NOD2, another NLR protein that can respond to bacterial muramyl dipeptide, to activate caspase-1 and induce IL-1β secretion, and this process was independent of ASC (100). In 2014, Van Opdenbosch N *et al.* discovered that, after treatment with LF, ASC-deficient murine macrophages produced IL-1β *via* NLRP1B (72). However, inflammasome signaling could be enhanced by speck formation induced by ASC, which then stimulated abundant amounts of caspase-1 to mediate optimum downstream responses, such as mature cytokine production (49). The detailed structure of NLRP1 oligomerization remains unclear because of the different locations of the transduction domain of NLRP1 compared with other inflammasomes (**Figure 2**).

Unlike hNLRP1, the N-terminus plays an essential role in the activation of mNLRP1B (62, 69). First, mNLRP1B undergoes N-terminal cleavage and generates a neo-N-terminus; then, it undergoes ubiquitination and full-length degradation induced by proteasomes *via* the N-end rule pathway. Subsequently, an effective inflammatory C-terminal fragment is released, with a UPA-like domain associated with CARD (77, 84). In favor of the CARD–CARD interaction, a simple inflammasome complex made up of the UPA–CARD fragment contains ASC and caspase-1. It is noteworthy that the activation of mNLRP1 takes place in the absence of the NOD domain, as it is removed *via* the N-end rule pathway, unlike hNLRP1, in which NOD constitutes the core of the inflammasome.

A high level of potassium is released into the extracellular environment after TBI, which stimulates pannexin-1 channels and leads to the recruitment of the NLRP1 inflammasome (54, 55, 101). Pannexin-1 mediates neurotoxic effects that can enhance plasma membrane permeability and stimulate inflammasomes (102). In addition, pannexin can be coactivated by P2X7, a type of ATP-gated cation channel that stimulates nonselective ionic passage, such as calcium efflux and potassium efflux, when activated by high levels of extracellular ATP. It has been reported that pannexin-1 is also involved in neuronal death induced by Aβ in Alzheimer’s disease and other neurodegenerative diseases (103–105). In addition, NLRP1 activation results in the cleavage of an antiapoptotic protein that is related to the caspase-invalidation-X-linked inhibitor of apoptosis protein (XIAP) (54, 106). The cleavage of XIAP leads to N-terminal BIR1–2 fragment production, which, in turn, further decreases the ability of XIAP to restrain caspases (107). Thus, the cleavage of XIAP during NLRP1 inflammasome activation may stimulate caspase-1 (107).

## NLRP1 as a Target for the Treatment of Brain Injury

### Subarachnoid Hemorrhage

SAH is one of the most common cerebrovascular diseases, and emerging evidence demonstrates that EBI following SAH plays a significant role in the poor outcomes of SAH (4, 5, 108). Among the potential mechanisms of EBI, such as apoptosis, autophagy, necroptosis, and neural death (3, 109–111), pyroptosis, which is a mechanism of programmed cell death, has emerged in recent decades (112). The formation of NLRP inflammasomes can transform pro-caspase-1 into its cleaved form, which induces the cleavage of pro-IL-1β and pro-IL-18, resulting in the inflammatory form of cell death known as pyroptosis (113).

In 2016, Wu Q *et al.* collected CSF from 24 SAH patients within 72 h of an attack and compared the samples with those from control patients who underwent artificial hip arthroplasty (12). The researchers found that the NLRP1, ASC, and caspase-1 levels were higher in the CSF of SAH patients than those of control patients and that elevated inflammasome protein levels showed a significant relationship with the severity and passive outcomes of SAH, along with acute hydrocephalus and CT cerebral oedema manifestations. The researchers also discovered that NLRP1 could act as an independent risk factor for SAH outcomes and ultimately concluded that the inflammasome proteins in the CSF of SAH patients could be biomarkers to evaluate EBI and poor clinical outcomes (12).

Pyroptosis is associated with various CNS diseases and is related to repair, aging, tumors, cerebral hemorrhage, and ischemia in the CNS (113–115). In 2021, Chen JH *et al.* found that atorvastatin, a cholesterol-lowering medicine, could dramatically improve the poor outcomes after SAH, such as the survival rates and neurological scores of mice and the survival rates of neurons. The outcomes were accompanied by the downregulation of NLRP1, cleaved caspase-1, IL-1β, and IL-18 expression (13). This discovery indicates a potential treatment for SAH by blocking pyroptosis and NLRP1 and NLRP3. Although atorvastatin has a promising effect on SAH, its practical application in patients requires further study. Whether other agents are beneficial for curing SAH *via* the NLRP1/caspase-1/IL-1β and IL-18 pathway or other inflammatory pathways remains unclear.

### Ischemic Brain Injury

Stroke is initiated by the rupture or obstruction of the cerebral vasculature, leading to brain injury, permanent disability, and even death (17). Ischemic stroke accounts for 80–85% of all stroke cases (ischemic and hemorrhagic stroke) worldwide, making it the most common type of stroke (116). A growing body of evidence suggests that inflammation in the immune system plays a significant role in this condition (117). Stimulation of the immune system upregulates proinflammatory cytokines, chemokines, and ROS, all of which cause neuroinflammation (118). Inflammasomes are strongly involved in the innate immune system inflammatory response (119).

The NLRP family of inflammasomes has been known to be closely associated with stroke pathogenesis. In regard to stroke, NLRP1 is increased and activates the inflammatory response, ultimately leading to neuronal death due to the decrease in Mi-R-9a-5p as a result of stroke (120). In an experiment with an ischemic stroke mouse model conducted by Dr. X. Sun, the NLRP2 expression in the treatment group was significantly higher than that in the control group (121). In addition, in 2015, Shabanzadeh AP *et al.* discovered that ischemic stroke could upregulate NLRP3 in neurons through the nuclear factor-kappa B (NF-κB) and mitogen-activated protein kinase (MAPK) pathways (122). Consistent with this finding, Fan DY *et al.* showed that NLRP1 and NLRP3 inflammasome proteins could be activated by stimulating the NF-κB or MAPK signaling pathways in cortical neurons and cerebral tissues *in vitro* and *in vivo*, and the inhibitors of these two pathways decrease the level of inflammation under ischemic conditions (24). In addition, the researchers showed that treatment with intravenous immunoglobulin could attenuate NF-κB and MAPK signaling pathway activation, leading to the suppression of NLRP1 and NLRP3 activation and enhancing the expression of BCL-2 and BCL-xL, both of which are antiapoptotic proteins (24).

Currently, propofol, an intravenous medicine that is widely used in surgical anesthesia and sedation (123), is becoming known for its neuroprotective effects. The mechanisms underlying its effects include the repression of proinflammatory factors, inhibition of apoptotic pathways, and stimulation of neuroprotective signaling (124, 125). In 2020, Ma Z *et al.* showed that oxygen-glucose deprivation could upregulated the NLRP1 and NLRP3 inflammasomes in ischemic cortical neurons; however, this process could be inhibited by propofol. The researchers concluded that propofol exerts its neuroprotective effects *via* the NLRP1–caspase-1–caspase-6 inflammatory pathway (126). As confirmed previously, caspase-6 is also closely associated with neurodegenerative diseases such as Huntington’s disease and Alzheimer’s disease (127). Whether propofol exerts beneficial effects on neurodegenerative diseases remains to be explored.

### Traumatic Brain Injury

The pathogenesis of TBI mainly consists of irreversible primary mechanical insult and multifactorial secondary brain injury, the effects of which may progress in the subsequent days or even years (26). Thus, secondary brain injury provides opportunities for therapeutic interventions in TBI (128). Many mechanisms are related to secondary injury, including neuroinflammation, calcium imbalance, oxidative stress, and apoptosis (129). Among these processes, neuroinflammation has often drawn more attention in studies exploring the progression of TBI (130). Generally, clearing cellular damage after injury and promoting neural repair call for a moderate inflammatory response, while extreme inflammation complicates neuronal insult and results in pathological deterioration (131). Thus, it is pivotal to control excessive inflammation during TBI treatment.

As an important factor in the induction and proliferation of inflammation, NLRP1 has attracted great interest in the neurotrauma field (132). de Rivero V *et al.* observed IL-1β cleavage, caspase-1 stimulation, XIAP cleavage, and increased NLRP1 inflammasome complex assembly in rats undergoing fluid percussion injury. The administration of anti-ASC-neutralizing antibodies could alleviate the inflammatory response and reduce the contusion volume (54). These studies demonstrated that the NLRP1 inflammasome plays a crucial role in the innate inflammatory response following TBI.

In 2012, de Rivero V *et al.* showed that, after trauma, the expression of the NLRP1 inflammasome, ASC, caspase-1, and IL-1β increased in the brain and spinal cord motor neurons (133). Furthermore, Satchell *et al.* reported an increase in caspase-1 and IL-1β protein expression in the CSF of infants and children who suffered severe TBI, which was associated with negative outcomes (134), and several years later, Adamczak S *et al.* observed the same effects in patients after severe or moderate TBI in a wider age range (17–65 years old), in which higher CSF levels of NLRP1, ASC, and caspase-1 were present than in the control group (33). In 2016, de Rivero V *et al.* discovered that exosomes derived from the CSF of patients suffering TBI and/or spinal cord injury contained NLRP1 inflammasome proteins (34). These exosomes play a critical role in the spread of inflammasomes in the CNS by exposing adjacent cells to their cargo proteins, such as IL-1β and inflammasome complexes (135).

In addition to the research conducted by V. de Rivero (54), in 2012, Tomura S *et al.* concluded that posttraumatic hypothermia treatment could effectively reduce P2X7 receptor levels, thus reducing the secretion of caspase-1 by neurons, suggesting a correlation between NLRP1 and P2X7 and a decrease in the innate immune response, ultimately demonstrating promising outcomes for rats after TBI (133). Despite the therapeutics mentioned previously, effective treatments for TBI patients targeting NLRP1 are lacking. With the increasing incidence of TBI, it is urgent to find a valid therapeutic strategy for treating TBI, and targeting NLRP1 and NLRP3 represents a promising strategy.

### Other Roles of NLRP1 in Brain Injury

In 2015, Wang YC *et al.* concluded that acid-sensing ion channels, which are channels that are significantly involved in pathophysiological CNS processes and can be stimulated by extracellular acidosis, are closely related to acidosis-induced cortical neuronal injury due to the activity of the NLRP1 inflammasome in the context of extracellular acidosis *via* the SIC-BK channel K+ signaling pathway (136).

It is well known that preterm infants can develop bronchopulmonary dysplasia, a type of chronic neonatal lung disease (137), which is often accompanied by an immature brain. Thus, these infants are prone to short- and/or long-term complications, such as intraventricular hemorrhage, brain paralysis, primary amentia, and cognitive deficits (138, 139). Studies have demonstrated that, in neonatal rats exposed to postnatal hyperoxia (85% O_2_), the NLRP1 inflammasome is stimulated in the lung and immature brain, resulting in inflammation, neurodegeneration (140–142), and even cell death (143). In 2019, Fredrick DS *et al.* conducted an experiment to explore hyperoxia-induced lung and brain injury in a neonatal rat model and found that hyperoxia induced NLRP1 inflammasome activation, resulting in alveolarization and vascular development in the lung along with cell death and a reduction in cell propagation in the subventricular zone (SVZ) and subgranular zone (SGZ) of the rat brain (144). As expected, the damage to the lung and brain caused by hyperoxia could be ameliorated by Ac-YVAD-CMK, an irreversible caspase-1 inhibitor, thus verifying that inhibiting caspase-1 reduced pyroptosis and facilitated cell proliferation in the SVZ and SGZ, providing a novel target for the treatment of lung and brain injury (144).

In addition, understanding the characteristics of NLRP1 could also be important for radiation-induced brain injury, but an obvious correlation between NLRP1 inflammasome activation and this kind of brain injury in the hippocampi of juvenile rats has not been found (145). It is worth noting that NLRP1 is a potential therapeutic target for brain injury, although experiments have provided conflicting results regarding the nonessential roles of NLRP1 in TBI in a murine model (55) and in the CSF of children and infants suffering from severe TBI (146). Interestingly, emerging evidence has emphasized the combined roles of NLRP1 and NLRP3 in the CNS (147, 148), especially in brain injury (24, 146, 149). Moreover, a report showed that high levels of NLRP1 and NLRP3 were present in the hippocampus of patients abusing methamphetamine (150), indicating the relationship between these two classic inflammasomes and a promising strategy for the treatment of brain injury.

## Conclusions and Future Perspectives

The NLRP3 inflammasome has been well studied in the progression of CNS diseases and is considered a predominant element in the inflammatory process. Here we view NLRP1-mediated inflammasome activation in neurons as equally important in neuroinflammation following brain injury. NLRP1 may be a therapeutic target for brain injury intervention because of its ability to induce inflammatory reactions. NLRP1 inhibitors have been reported to hinder the formation of the inflammasome complex and ATP binding (151). Hence, it is possible to use NLRP1 inhibitors as novel anti-inflammatory drugs to relieve neuronal damage and moderate the progression of disease in brain injury patients. Hopefully, this treatment strategy may benefit patients affected by CNS diseases. However, the correlation between NLRP1 and NLRP3 in the CNS warrants further investigation. Neuroinflammation is collectively regarded as the basic cause of neuronal insult in brain injury.

To advance the creation of a promising cure for brain injury, it is necessary to fully understand how the NLRP1 inflammasome operates in the brain under both physiological and pathological conditions. However, due to the sophistication of the NLRP1 inflammasome and the differences between human and mouse orthologs, there are many challenges in applying our experimental results to clinical research, demonstrating that the treatment of brain injury patients by targeting NLRP1 remains insufficient. The coordination of basic researchers and clinicians will shed light on the underlying key mechanisms of inflammation and transfer this knowledge from bench to bedside.

## Author Contributions

LM, XM, and YC wrote the manuscript. LM created the figures. JZ approved the final version of the manuscript. XC provided overall guidance. All authors contributed to the article and approved the submitted version.

## Funding

This work was supported by grants from the National Natural Science Foundation of China (numbers 816719029 and 81370029), the Project of Tianjin Applied Basic and Cutting-Edge Technological Research (number 17JCYBJC25200), the Scientific Research Programme of Tianjin Education Commission (Natural Science) of China (number 2019ZD034), and the Tianjin Health Care Elite Prominent Young Doctor Development Program and the Young and Middle-Aged Backbone Innovative Talent Program.

## Conflict of Interest

The authors declare that this research was conducted in the absence of any commercial or financial relationships that could be construed as a potential conflict of interest.

## Publisher’s Note

All claims expressed in this article are solely those of the authors and do not necessarily represent those of their affiliated organizations, or those of the publisher, the editors and the reviewers. Any product that may be evaluated in this article, or claim that may be made by its manufacturer, is not guaranteed or endorsed by the publisher.

## References

[1] Fan H Ding R Liu W Zhang X Li R Wei B. Heat Shock Protein 22 Modulates NRF1/TFAM-Dependent Mitochondrial Biogenesis and DRP1-Sparked Mitochondrial Apoptosis Through AMPK-Pgc1α Signaling Pathway to Alleviate the Early Brain Injury of Subarachnoid Hemorrhage in Rats. Redox Biol (2021) 40:101856. doi: 10.1016/j.redox.2021.101856 33472123 PMC7816003

[2] Macdonald RL Schweizer TA. Spontaneous Subarachnoid Haemorrhage. Lancet (London England) (2017) 389:655–66. doi: 10.1016/S0140-6736(16)30668-7 27637674

[3] Chen J Li M Zhu X Chen L Yang S Zhang C. Atorvastatin Reduces Cerebral Vasospasm and Infarction After Aneurysmal Subarachnoid Hemorrhage in Elderly Chinese Adults. Aging (2020) 12:2939–51. doi: 10.18632/aging.102788 PMC7041764 32035420

[4] Chen J Xuan Y Chen Y Wu T Chen L Guan H. Netrin-1 Alleviates Subarachnoid Haemorrhage-Induced Brain Injury *via* the Pparγ/NF-KB Signalling Pathway. J Cell Mol Med (2019) 23:2256–62. doi: 10.1111/jcmm.14105 PMC6378208 30614619

[5] Chen JH Wu T Yang LK Chen L Zhu J Li PP. Protective Effects of Atorvastatin on Cerebral Vessel Autoregulation in an Experimental Rabbit Model of Subarachnoid Hemorrhage. Mol Med Rep (2018) 17:1651–9. doi: 10.3892/mmr.2017.8074 PMC5780106 29257200

[6] Cahill J Cahill WJ Calvert JW Calvert JH Zhang JH. Mechanisms of Early Brain Injury After Subarachnoid Hemorrhage. J Cereb Blood Flow Metab (2006) 26:1341–53. doi: 10.1038/sj.jcbfm.9600283 16482081

[7] Fan LF He PY Peng YC Du QH Ma YJ Jin JX. Mdivi-1 Ameliorates Early Brain Injury After Subarachnoid Hemorrhage *via* the Suppression of Inflammation-Related Blood-Brain Barrier Disruption and Endoplasmic Reticulum Stress-Based Apoptosis. Free Radical Biol Med (2017) 112:336–49. doi: 10.1016/j.freeradbiomed.2017.08.003 28790012

[8] Galluzzi L Bravo-San Pedro JM Blomgren K Kroemer G. Autophagy in Acute Brain Injury. Nat Rev Neurosci (2016) 17:467–84. doi: 10.1038/nrn.2016.51 27256553

[9] Mo J Enkhjargal B Travis ZD Zhou K Wu P Zhang G. AVE 0991 Attenuates Oxidative Stress and Neuronal Apoptosis *via* Mas/PKA/CREB/UCP-2 Pathway After Subarachnoid Hemorrhage in Rats. Redox Biol (2019) 20:75–86. doi: 10.1016/j.redox.2018.09.022 30296700 PMC6174866

[10] Zhang K Kaufman RJ. From Endoplasmic-Reticulum Stress to the Inflammatory Response. Nature (2008) 454:455–62. doi: 10.1038/nature07203 PMC2727659 18650916

[11] Xu P Tao C Zhu Y Wang G Kong L Li W. TAK1 Mediates Neuronal Pyroptosis in Early Brain Injury After Subarachnoid Hemorrhage. J Neuroinflamm (2021) 18:188. doi: 10.1186/s12974-021-02226-8 PMC8406585 34461942

[12] Wu Q Wang XL Yu Q Pan H Zhang XS Zhang QR. Inflammasome Proteins in Cerebrospinal Fluid of Patients With Subarachnoid Hemorrhage are Biomarkers of Early Brain Injury and Functional Outcome. World Neurosurg (2016) 94:472–9. doi: 10.1016/j.wneu.2016.07.039 27443226

[13] Chen J Zhang C Yan T Yang L Wang Y Shi Z. Atorvastatin Ameliorates Early Brain Injury After Subarachnoid Hemorrhage *via* Inhibition of Pyroptosis and Neuroinflammation. J Cell Physiol (2021) 236(10):6920–31. doi: 10.1002/jcp.30351 33792028

[14] Ahmad M Graham SH. Inflammation After Stroke: Mechanisms and Therapeutic Approaches. Trans Stroke Res (2010) 1:74–84. doi: 10.1007/s12975-010-0023-7 PMC2956985 20976117

[15] Gilgun-Sherki Y Rosenbaum Z Melamed E Offen D. Antioxidant Therapy in Acute Central Nervous System Injury: Current State. Pharmacol Rev (2002) 54:271–84. doi: 10.1124/pr.54.2.271 12037143

[16] Bacigaluppi M Comi G Hermann DM. Animal Models of Ischemic Stroke. Part Two: Modeling Cerebral Ischemia. Open Neurol J (2010) 4:34–8. doi: 10.2174/1874205X01004010034 PMC2923341 20721320

[17] Durukan A Tatlisumak T. Acute Ischemic Stroke: Overview of Major Experimental Rodent Models, Pathophysiology, and Therapy of Focal Cerebral Ischemia. Pharmacol Biochem Behav (2007) 87:179–97. doi: 10.1016/j.pbb.2007.04.015 17521716

[18] Kumar G Goyal MK Sahota PK Jain R. Penumbra, the Basis of Neuroimaging in Acute Stroke Treatment: Current Evidence. J Neurol Sci (2010) 288:13–24. doi: 10.1016/j.jns.2009.09.027 19875134

[19] Alawieh A Langley EF Tomlinson S. Targeted Complement Inhibition Salvages Stressed Neurons and Inhibits Neuroinflammation After Stroke in Mice. Sci Trans Med (2018) 10:42. doi: 10.1126/scitranslmed.aao6459 PMC6689196 29769288

[20] DiSabato DJ Quan N Godbout JP. Neuroinflammation: The Devil is in the Details. J Neurochem (2016), 139:136–53. doi: 10.1111/jnc.13607 PMC5025335 26990767

[21] Xu Q Zhao B Ye Y Li Y Zhang Y Xiong X. Relevant Mediators Involved in and Therapies Targeting the Inflammatory Response Induced by Activation of the NLRP3 Inflammasome in Ischemic Stroke. J Neuroinflamm (2021) 18:123. doi: 10.1186/s12974-021-02137-8 PMC8166383 34059091

[22] Chen W Guo C Feng H Chen Y. Mitochondria: Novel Mechanisms and Therapeutic Targets for Secondary Brain Injury After Intracerebral Hemorrhage. Front Aging Neurosci (2020) 12:615451. doi: 10.3389/fnagi.2020.615451 33584246 PMC7873050

[23] Forrester SJ Kikuchi DS Hernandes MS Xu Q Griendling KK. Reactive Oxygen Species in Metabolic and Inflammatory Signaling. Circ Res (2018) 122:877–902. doi: 10.1161/CIRCRESAHA.117.311401 29700084 PMC5926825

[24] Fann DY Lim YA Cheng YL Lok KZ Chunduri P Baik SH. Evidence That NF-κB and MAPK Signaling Promotes NLRP Inflammasome Activation in Neurons Following Ischemic Stroke. Mol Neurobiol (2018) 55:1082–96. doi: 10.1007/s12035-017-0394-9 28092085

[25] Fann DY Lee SY Manzanero S Tang SC Gelderblom M Chunduri P. Intravenous Immunoglobulin Suppresses NLRP1 and NLRP3 Inflammasome-Mediated Neuronal Death in Ischemic Stroke. Cell Death Dis (2013) 4:e790. doi: 10.1038/cddis.2013.326 24008734 PMC3789184

[26] Garcia JM Lopez-Rodriguez AB. Editorial: Neuroendocrine Disorders After Traumatic Brain Injury: Past, Present and Future. Front Endocrinol (2019) 10:386. doi: 10.3389/fendo.2019.00386 PMC6607278 31297087

[27] Lozano D Gonzales-Portillo GS Acosta S de la Pena I Tajiri N Kaneko Y. Neuroinflammatory Responses to Traumatic Brain Injury: Etiology, Clinical Consequences, and Therapeutic Opportunities. Neuropsychiatr Dis Treat (2015) 11:97–106. doi: 10.2147/NDT.S65815 25657582 PMC4295534

[28] Chodobski A Zink BJ Szmydynger-Chodobska J. Blood-Brain Barrier Pathophysiology in Traumatic Brain Injury. Trans Stroke Res (2011) 2:492–516. doi: 10.1007/s12975-011-0125-x PMC3268209 22299022

[29] Dasuri K Zhang L Keller JN. Oxidative Stress, Neurodegeneration, and the Balance of Protein Degradation and Protein Synthesis. Free Radical Biol Med (2013) 62:170–85. doi: 10.1016/j.freeradbiomed.2012.09.016 23000246

[30] Liu HD Li W Chen ZR Hu YC Zhang DD Shen W. Expression of the NLRP3 Inflammasome in Cerebral Cortex After Traumatic Brain Injury in a Rat Model. Neurochem Res (2013) 38:2072–83. doi: 10.1007/s11064-013-1115-z 23892989

[31] Adamczak SE de Rivero Vaccari JP Dale G Brand FJ Nonner D Bullock MR. Pyroptotic Neuronal Cell Death Mediated by the AIM2 Inflammasome. J Cereb Blood Flow Metab (2014) 34:621–9. doi: 10.1038/jcbfm.2013.236 PMC3982080 24398937

[32] Campello Yurgel V Ikuta N Brondani da Rocha A Lunge VR Fett Schneider R Kazantzi Fonseca AS. Role of Plasma DNA as a Predictive Marker of Fatal Outcome Following Severe Head Injury in Males. J Neurotrauma (2007) 24:1172–81. doi: 10.1089/neu.2006.0160 17610356

[33] Adamczak S Dale G de Rivero Vaccari JP Bullock MR Dietrich WD Keane RW. Inflammasome Proteins in Cerebrospinal Fluid of Brain-Injured Patients as Biomarkers of Functional Outcome: Clinical Article. J Neurosurg (2012) 117:1119–25. doi: 10.3171/2012.9.JNS12815 PMC3576729 23061392

[34] de Rivero Vaccari JP Brand F Adamczak S Lee SW Perez-Barcena J Wang MY. Exosome-Mediated Inflammasome Signaling After Central Nervous System Injury. J Neurochem (2016) 136:39–48. doi: 10.1111/jnc.13036 25628216 PMC4516699

[35] Vance RE Isberg RR Portnoy DA. Patterns of Pathogenesis: Discrimination of Pathogenic and Nonpathogenic Microbes by the Innate Immune System. Cell Host Microbe (2009) 6:10–21. doi: 10.1016/j.chom.2009.06.007 19616762 PMC2777727

[36] Akar-Ghibril N. Defects of the Innate Immune System and Related Immune Deficiencies. Clin Rev Allergy Immunol (2021). doi: 10.1007/s12016-021-08885-y 34417936

[37] Takeuchi O Akira S. Pattern Recognition Receptors and Inflammation. Cell (2010) 140:805–20. doi: 10.1016/j.cell.2010.01.022 20303872

[38] Griffiths MR Gasque P Neal JW. The Regulation of the CNS Innate Immune Response is Vital for the Restoration of Tissue Homeostasis (Repair) After Acute Brain Injury: A Brief Review. Int J Inflam (2010) 2010:151097. doi: 10.4061/2010/151097 21152121 PMC2989866

[39] Hauwel M Furon E Canova C Griffiths M Neal J Gasque P. Innate (Inherent) Control of Brain Infection, Brain Inflammation and Brain Repair: The Role of Microglia, Astrocytes, "Protective" Glial Stem Cells and Stromal Ependymal Cells. Brain Res Rev (2005) 48:220–33. doi: 10.1016/j.brainresrev.2004.12.012 15850661

[40] Daneman R Prat A. The Blood-Brain Barrier. Cold Spring Harb Perspect Biol (2015) 7:a020412. doi: 10.1101/cshperspect.a020412 25561720 PMC4292164

[41] Lafon M Megret F Lafage M Prehaud C. The Innate Immune Facet of Brain: Human Neurons Express TLR-3 and Sense Viral dsRNA. J Mol Neurosci (2006) 29:185–94. doi: 10.1385/JMN:29:3:185 17085778

[42] Ransohoff RM Brown MA. Innate Immunity in the Central Nervous System. J Clin Invest (2012) 122:1164–71. doi: 10.1172/JCI58644 PMC3314450 22466658

[43] Zendedel A Mönnink F Hassanzadeh G Zaminy A Ansar MM Habib P. Estrogen Attenuates Local Inflammasome Expression and Activation After Spinal Cord Injury. Mol Neurobiol (2018) 55:1364–75. doi: 10.1007/s12035-017-0400-2 28127698

[44] Pedra JH Cassel SL Sutterwala FS. Sensing Pathogens and Danger Signals by the Inflammasome. Curr Opin Immunol (2009) 21:10–6. doi: 10.1016/j.coi.2009.01.006 PMC2701640 19223160

[45] de Rivero Vaccari JP Dietrich WD Keane RW. Therapeutics Targeting the Inflammasome After Central Nervous System Injury. Trans Res (2016) 167:35–45. doi: 10.1016/j.trsl.2015.05.003 PMC4643411 26024799

[46] Broz P Dixit VM. Inflammasomes: Mechanism of Assembly, Regulation and Signalling. Nat Rev Immunol (2016) 16:407–20. doi: 10.1038/nri.2016.58 27291964

[47] Cai X Chen J Xu H Liu S Jiang QX Halfmann R. Prion-Like Polymerization Underlies Signal Transduction in Antiviral Immune Defense and Inflammasome Activation. Cell (2014) 156:1207–22. doi: 10.1016/j.cell.2014.01.063 PMC4034535 24630723

[48] Lu A Magupalli VG Ruan J Yin Q Atianand MK Vos MR. Unified Polymerization Mechanism for the Assembly of ASC-Dependent Inflammasomes. Cell (2014) 156:1193–206. doi: 10.1016/j.cell.2014.02.008 PMC4000066 24630722

[49] Dick MS Sborgi L Rühl S Hiller S Broz P. ASC Filament Formation Serves as a Signal Amplification Mechanism for Inflammasomes. Nat Commun (2016) 7:11929. doi: 10.1038/ncomms11929 27329339 PMC4917984

[50] Lamkanfi M Dixit VM. Mechanisms and Functions of Inflammasomes. Cell (2014) 157:1013–22. doi: 10.1016/j.cell.2014.04.007 24855941

[51] Platnich JM Muruve DA. NOD-Like Receptors and Inflammasomes: A Review of Their Canonical and non-Canonical Signaling Pathways. Arch Biochem Biophys (2019) 670:4–14. doi: 10.1016/j.abb.2019.02.008 30772258

[52] Kummer JA Broekhuizen R Everett H Agostini L Kuijk L Martinon F. Inflammasome Components NALP 1 and 3 Show Distinct But Separate Expression Profiles in Human Tissues Suggesting a Site-Specific Role in the Inflammatory Response. J Histochem Cytochem (2007) 55:443–52. doi: 10.1369/jhc.6A7101.2006 17164409

[53] Masters SL Gerlic M Metcalf D Preston S Pellegrini M O'Donnell JA. NLRP1 Inflammasome Activation Induces Pyroptosis of Hematopoietic Progenitor Cells. Immunity (2012) 37:1009–23. doi: 10.1016/j.immuni.2012.08.027 PMC4275304 23219391

[54] de Rivero Vaccari JP Lotocki G Alonso OF Bramlett HM Dietrich WD Keane RW. Therapeutic Neutralization of the NLRP1 Inflammasome Reduces the Innate Immune Response and Improves Histopathology After Traumatic Brain Injury. J Cereb Blood Flow Metab (2009) 29:1251–61. doi: 10.1038/jcbfm.2009.46 PMC2846547 19401709

[55] Brickler T Gresham K Meza A Coutermarsh-Ott S Williams TM Rothschild DE. Nonessential Role for the NLRP1 Inflammasome Complex in a Murine Model of Traumatic Brain Injury. Mediators Inflamm (2016) 2016:6373506. doi: 10.1155/2016/6373506 27199506 PMC4854993

[56] Mawhinney L de Rivero Vaccari J Dale G Keane R Bramlett H. Heightened Inflammasome Activation is Linked to Age-Related Cognitive Impairment in Fischer 344 Rats. BMC Neurosci (2011) 12:123. doi: 10.1186/1471-2202-12-123 22133203 PMC3259063

[57] Sagulenko V Thygesen SJ Sester DP Idris A Cridland JA Vajjhala PR. AIM2 and NLRP3 Inflammasomes Activate Both Apoptotic and Pyroptotic Death Pathways *via* ASC. Cell Death Differ (2013) 20:1149–60. doi: 10.1038/cdd.2013.37 PMC3741496 23645208

[58] Barrington J Lemarchand E Allan SM. A Brain in Flame; do Inflammasomes and Pyroptosis Influence Stroke Pathology? Brain Pathol (Zurich Switzerland) (2017) 27:205–12. doi: 10.1111/bpa.12476 PMC8028888 27997059

[59] Inohara Chamaillard McDonald C Nuñez G. NOD-LRR Proteins: Role in Host-Microbial Interactions and Inflammatory Disease. Annu Rev Biochem (2005) 74:355–83. doi: 10.1146/annurev.biochem.74.082803.133347 15952891

[60] Kersse K Bertrand MJ Lamkanfi M Vandenabeele P. NOD-Like Receptors and the Innate Immune System: Coping With Danger, Damage and Death. Cytokine Growth Factor Rev (2011) 22:257–76. doi: 10.1016/j.cytogfr.2011.09.003 21996492

[61] D'Osualdo A Weichenberger CX Wagner RN Godzik A Wooley J Reed JC. CARD8 and NLRP1 Undergo Autoproteolytic Processing Through a ZU5-Like Domain. PloS One (2011) 6:e27396. doi: 10.1371/journal.pone.0027396 22087307 PMC3210808

[62] Finger JN Lich JD Dare LC Cook MN Brown KK Duraiswami C. Autolytic Proteolysis Within the Function to Find Domain (FIIND) is Required for NLRP1 Inflammasome Activity. J Biol Chem (2012) 287:25030–7. doi: 10.1074/jbc.M112.378323 PMC3408201 22665479

[63] Frew BC Joag VR Mogridge J. Proteolytic Processing of Nlrp1b is Required for Inflammasome Activity. PloS Pathog (2012) 8:e1002659. doi: 10.1371/journal.ppat.1002659 22536155 PMC3334886

[64] Maharana J. Elucidating the Interfaces Involved in CARD-CARD Interactions Mediated by NLRP1 and Caspase-1 Using Molecular Dynamics Simulation. J Mol Graphics Model (2018) 80:7–14. doi: 10.1016/j.jmgm.2017.12.016 29324327

[65] Park HH Lo YC Lin SC Wang L Yang JK Wu H. The Death Domain Superfamily in Intracellular Signaling of Apoptosis and Inflammation. Annu Rev Immunol (2007) 25:561–86. doi: 10.1146/annurev.immunol.25.022106.141656 PMC2904440 17201679

[66] Broz P von Moltke J Jones JW Vance RE Monack DM. Differential Requirement for Caspase-1 Autoproteolysis in Pathogen-Induced Cell Death and Cytokine Processing. Cell Host Microbe (2010) 8:471–83. doi: 10.1016/j.chom.2010.11.007 PMC3016200 21147462

[67] Mariathasan S Newton K Monack DM Vucic D French DM Lee WP. Differential Activation of the Inflammasome by Caspase-1 Adaptors ASC and Ipaf. Nature (2004) 430:213–8. doi: 10.1038/nature02664 15190255

[68] Chavarría-Smith J Vance RE. Direct Proteolytic Cleavage of NLRP1B is Necessary and Sufficient for Inflammasome Activation by Anthrax Lethal Factor. PloS Pathog (2013) 9:e1003452. doi: 10.1371/journal.ppat.1003452 23818853 PMC3688554

[69] Chavarría-Smith J Mitchell PS Ho AM Daugherty MD Vance RE. Functional and Evolutionary Analyses Identify Proteolysis as a General Mechanism for NLRP1 Inflammasome Activation. PloS Pathog (2016) 12:e1006052. doi: 10.1371/journal.ppat.1006052 27926929 PMC5142783

[70] Boyden ED Dietrich WF. Nalp1b Controls Mouse Macrophage Susceptibility to Anthrax Lethal Toxin. Nat Genet (2006) 38:240–4. doi: 10.1038/ng1724 16429160

[71] Sastalla I Crown D Masters SL McKenzie A Leppla SH Moayeri M. Transcriptional Analysis of the Three Nlrp1 Paralogs in Mice. BMC Genomics (2013) 14:188. doi: 10.1186/1471-2164-14-188 23506131 PMC3641005

[72] Van Opdenbosch N Gurung P Vande Walle L Fossoul A Kanneganti TD Lamkanfi M. Activation of the NLRP1b Inflammasome Independently of ASC-Mediated Caspase-1 Autoproteolysis and Speck Formation. Nat Commun (2014) 5:3209. doi: 10.1038/ncomms4209 24492532 PMC3926011

[73] Gai K Okondo MC Rao SD Chui AJ Ball DP Johnson DC. DPP8/9 Inhibitors are Universal Activators of Functional NLRP1 Alleles. Cell Death Dis (2019) 10:587. doi: 10.1038/s41419-019-1817-5 31383852 PMC6683174

[74] Newman ZL Printz MP Liu S Crown D Breen L Miller-Randolph S. Susceptibility to Anthrax Lethal Toxin-Induced Rat Death is Controlled by a Single Chromosome 10 Locus That Includes Rnlrp1. PloS Pathog (2010) 6:e1000906. doi: 10.1371/journal.ppat.1000906 20502689 PMC2873920

[75] Chavarría-Smith J Vance RE. The NLRP1 Inflammasomes. Immunol Rev (2015) 265:22–34. doi: 10.1111/imr.12283 25879281

[76] Hellmich KA Levinsohn JL Fattah R Newman ZL Maier N Sastalla I. Anthrax Lethal Factor Cleaves Mouse Nlrp1b in Both Toxin-Sensitive and Toxin-Resistant Macrophages. PloS One (2012) 7:e49741. doi: 10.1371/journal.pone.0049741 23152930 PMC3495862

[77] Sandstrom A Mitchell PS Goers L Mu EW Lesser CF Vance RE. Functional Degradation: A Mechanism of NLRP1 Inflammasome Activation by Diverse Pathogen Enzymes. Science (New York N Y) (2019) 364. doi: 10.1126/science.aau1330 PMC6532986 30872533

[78] Cirelli KM Gorfu G Hassan MA Printz M Crown D Leppla SH. Inflammasome Sensor NLRP1 Controls Rat Macrophage Susceptibility to Toxoplasma Gondii. PloS Pathog (2014) 10:e1003927. doi: 10.1371/journal.ppat.1003927 24626226 PMC3953412

[79] Cavailles P Flori P Papapietro O Bisanz C Lagrange D Pilloux L. A Highly Conserved Toxo1 Haplotype Directs Resistance to Toxoplasmosis and its Associated Caspase-1 Dependent Killing of Parasite and Host Macrophage. PloS Pathog (2014) 10:e1004005. doi: 10.1371/journal.ppat.1004005 24699513 PMC3974857

[80] Liao KC Mogridge J. Activation of the Nlrp1b Inflammasome by Reduction of Cytosolic ATP. Infect Immun (2013) 81:570–9. doi: 10.1128/IAI.01003-12 PMC3553809 23230290

[81] Neiman-Zenevich J Liao KC Mogridge J. Distinct Regions of NLRP1B are Required to Respond to Anthrax Lethal Toxin and Metabolic Inhibition. Infect Immun (2014) 82:3697–703. doi: 10.1128/IAI.02167-14 PMC4187829 24935976

[82] Roberts JE Watters JW Ballard JD Dietrich WF. Ltx1, a Mouse Locus That Influences the Susceptibility of Macrophages to Cytolysis Caused by Intoxication With Bacillus Anthracis Lethal Factor, Maps to Chromosome 11. Mol Microbiol (1998) 29:581–91. doi: 10.1046/j.1365-2958.1998.00953.x 9720874

[83] Moayeri M Sastalla I Leppla SH. Anthrax and the Inflammasome. Microbes Infect (2012) 14:392–400. doi: 10.1016/j.micinf.2011.12.005 22207185 PMC3322314

[84] Chui AJ Okondo MC Rao SD Gai K Griswold AR Johnson DC. N-Terminal Degradation Activates the NLRP1B Inflammasome. Science (New York N Y) (2019) 364:82–5. doi: 10.1126/science.aau1208 PMC6610862 30872531

[85] Varshavsky A. The N-End Rule Pathway and Regulation by Proteolysis. Protein Sci (2011) 20:1298–345. doi: 10.1002/pro.666 PMC3189519 21633985

[86] Sriram SM Kim BY Kwon YT. The N-End Rule Pathway: Emerging Functions and Molecular Principles of Substrate Recognition. Nat Rev Mol Cell Biol (2011) 12:735–47. doi: 10.1038/nrm3217 22016057

[87] Xu H Shi J Gao H Liu Y Yang Z Shao F. The N-End Rule Ubiquitin Ligase UBR2 Mediates NLRP1B Inflammasome Activation by Anthrax Lethal Toxin. EMBO J (2019) 38:e101996. doi: 10.15252/embj.2019101996 31268597 PMC6600268

[88] Robinson K Teo D Tan K Toh G Ong H Lim C. Enteroviral 3C Protease Activates the Human NLRP1 Inflammasome in Airway Epithelia. Science (New York N Y) (2020) 370:1182. doi: 10.1126/science.aay2002 33093214

[89] Mitchell PS Sandstrom A Vance RE. The NLRP1 Inflammasome: New Mechanistic Insights and Unresolved Mysteries. Curr Opin Immunol (2019) 60:37–45. doi: 10.1016/j.coi.2019.04.015 31121538 PMC6800612

[90] Gov L Karimzadeh A Ueno N Lodoen MB. Human Innate Immunity to Toxoplasma Gondii is Mediated by Host Caspase-1 and ASC and Parasite GRA15. mBio (2013) 4. doi: 10.1128/mBio.00255-13 PMC3705447 23839215

[91] Bachovchin DA Koblan LW Wu W Liu Y Li Y Zhao P. A High-Throughput, Multiplexed Assay for Superfamily-Wide Profiling of Enzyme Activity. Nat Chem Biol (2014) 10:656–63. doi: 10.1038/nchembio.1578 PMC5953424 24997602

[92] Okondo MC Johnson DC Sridharan R Go EB Chui AJ Wang MS. DPP8 and DPP9 Inhibition Induces Pro-Caspase-1-Dependent Monocyte and Macrophage Pyroptosis. Nat Chem Biol (2017) 13:46–53. doi: 10.1038/nchembio.2229 27820798 PMC5477230

[93] Okondo MC Rao SD Taabazuing CY Chui AJ Poplawski SE Johnson DC. Inhibition of Dpp8/9 Activates the Nlrp1b Inflammasome. Cell Chem Biol (2018) 25:262–267.e5. doi: 10.1016/j.chembiol.2017.12.013 29396289 PMC5856610

[94] Zhong FL Robinson K Teo DET Tan KY Lim C Harapas CR. Human DPP9 Represses NLRP1 Inflammasome and Protects Against Autoinflammatory Diseases *via* Both Peptidase Activity and FIIND Domain Binding. J Biol Chem (2018) 293:18864–78. doi: 10.1074/jbc.RA118.004350 PMC6295727 30291141

[95] Johnson DC Taabazuing CY Okondo MC Chui AJ Rao SD Brown FC. DPP8/DPP9 Inhibitor-Induced Pyroptosis for Treatment of Acute Myeloid Leukemia. Nat Med (2018) 24:1151–6. doi: 10.1038/s41591-018-0082-y PMC6082709 29967349

[96] Neiman-Zenevich J Stuart S Abdel-Nour M Girardin SE Mogridge J. Listeria Monocytogenes and Shigella Flexneri Activate the NLRP1B Inflammasome. Infect Immun (2017) 85. doi: 10.1128/IAI.00338-17 PMC5649023 28808162

[97] Bachovchin DA Cravatt BF. The Pharmacological Landscape and Therapeutic Potential of Serine Hydrolases. Nat Rev Drug Discov (2012) 11:52–68. doi: 10.1038/nrd3620 22212679 PMC3665514

[98] Blume M Seeber F. Toxoplasma Gondiimetabolic Interactions Between and its Host. F1000Res (2018) 7. doi: 10.12688/f1000research.16021.1 PMC6208699 30467519

[99] Faustin B Lartigue L Bruey JM Luciano F Sergienko E Bailly-Maitre B. Reconstituted NALP1 Inflammasome Reveals Two-Step Mechanism of Caspase-1 Activation. Mol Cell (2007) 25:713–24. doi: 10.1016/j.molcel.2007.01.032 17349957

[100] Hsu L Ali S McGillivray S Tseng P Mariathasan S Humke E. A NOD2-NALP1 Complex Mediates Caspase-1-Dependent IL-1beta Secretion in Response to Bacillus Anthracis Infection and Muramyl Dipeptide. Proc Natl Acad Sci U S A (2008) 105:7803–8. doi: 10.1073/pnas.0802726105 PMC2409384 18511561

[101] Pétrilli V Papin S Dostert C Mayor A Martinon F Tschopp J. Activation of the NALP3 Inflammasome is Triggered by Low Intracellular Potassium Concentration. Cell Death Differ (2007) 14:1583–9. doi: 10.1038/sj.cdd.4402195 17599094

[102] Shestopalov VI Slepak VZ. Molecular Pathways of Pannexin1-Mediated Neurotoxicity. Front Physiol (2014) 5:23. doi: 10.3389/fphys.2014.00023 24575045 PMC3920106

[103] Sáez-Orellana F Godoy PA Bastidas CY Silva-Grecchi T Guzmán L Aguayo LG. ATP Leakage Induces P2XR Activation and Contributes to Acute Synaptic Excitotoxicity Induced by Soluble Oligomers of β-Amyloid Peptide in Hippocampal Neurons. Neuropharmacology (2016) 100:116–23. doi: 10.1016/j.neuropharm.2015.04.005 25896766

[104] Sáez-Orellana F Fuentes-Fuentes MC Godoy PA Silva-Grecchi T Panes JD Guzmán L. P2X Receptor Overexpression Induced by Soluble Oligomers of Amyloid Beta Peptide Potentiates Synaptic Failure and Neuronal Dyshomeostasis in Cellular Models of Alzheimer's Disease. Neuropharmacology (2018) 128:366–78. doi: 10.1016/j.neuropharm.2017.10.027 PMC5858180 29079292

[105] Yun SP Kam TI Panicker N Kim S Oh Y Park JS. Block of A1 Astrocyte Conversion by Microglia is Neuroprotective in Models of Parkinson's Disease. Nat Med (2018) 24:931–8. doi: 10.1038/s41591-018-0051-5 PMC6039259 29892066

[106] Katz LM Lotocki G Wang Y Kraydieh S Dietrich WD Keane RW. Regulation of Caspases and XIAP in the Brain After Asphyxial Cardiac Arrest in Rats. Neuroreport (2001) 12:3751–4. doi: 10.1097/00001756-200112040-00029 11726787

[107] de Rivero Vaccari JP Lotocki G Marcillo AE Dietrich WD Keane RW. A Molecular Platform in Neurons Regulates Inflammation After Spinal Cord Injury. J Neurosci (2008) 28:3404–14. doi: 10.1523/JNEUROSCI.0157-08.2008 PMC6670583 18367607

[108] Chen JH Wu T Xia WY Shi ZH Zhang CL Chen L. An Early Neuroprotective Effect of Atorvastatin Against Subarachnoid Hemorrhage. Neural Regen Res (2020) 15:1947–54. doi: 10.4103/1673-5374.280326 PMC7513987 32246644

[109] Cahill J Zhang JH. Subarachnoid Hemorrhage: Is it Time for a New Direction? Stroke (2009) 40:S86–7. doi: 10.1161/STROKEAHA.108.533315 PMC2743545 19064787

[110] Dong Y Fan C Hu W Jiang S Ma Z Yan X. Melatonin Attenuated Early Brain Injury Induced by Subarachnoid Hemorrhage *via* Regulating NLRP3 Inflammasome and Apoptosis Signaling. J Pineal Res (2016) 60:253–62. doi: 10.1111/jpi.12300 26639408

[111] Kenny EM Fidan E Yang Q Anthonymuthu TS New LA Meyer EA. Ferroptosis Contributes to Neuronal Death and Functional Outcome After Traumatic Brain Injury. Crit Care Med (2019) 47:410–8. doi: 10.1097/CCM.0000000000003555 PMC6449247 30531185

[112] Fricker M Tolkovsky AM Borutaite V Coleman M Brown GC. Neuronal Cell Death. Physiol Rev (2018) 98:813–80. doi: 10.1152/physrev.00011.2017 PMC5966715 29488822

[113] Huang J Lu W Doycheva DM Gamdzyk M Hu X Liu R. Ire1α Inhibition Attenuates Neuronal Pyroptosis *via* miR-125/NLRP1 Pathway in a Neonatal Hypoxic-Ischemic Encephalopathy Rat Model. J Neuroinflamm (2020) 17:152. doi: 10.1186/s12974-020-01796-3 PMC7203836 32375838

[114] Li Q Cao Y Dang C Han B Han R Ma H. Inhibition of Double-Strand DNA-Sensing cGAS Ameliorates Brain Injury After Ischemic Stroke. EMBO Mol Med (2020) 12:e11002. doi: 10.15252/emmm.201911002 32239625 PMC7136961

[115] Sun Z Nyanzu M Yang S Zhu X Wang K Ru J. κVX765 Attenuates Pyroptosis and HMGB1/TLR4/NF-B Pathways to Improve Functional Outcomes in TBI Mice. Oxid Med Cell Longev (2020) 2020:7879629. doi: 10.1155/2020/7879629 32377306 PMC7181015

[116] Zhang GL Zhu ZH Wang YZ. Neural Stem Cell Transplantation Therapy for Brain Ischemic Stroke: Review and Perspectives. World J Stem Cells (2019) 11:817–30. doi: 10.4252/wjsc.v11.i10.817 PMC6828598 31692854

[117] Jiang C Kong W Wang Y Ziai W Yang Q Zuo F. Changes in the Cellular Immune System and Circulating Inflammatory Markers of Stroke Patients. Oncotarget (2017) 8:3553–67. doi: 10.18632/oncotarget.12201 PMC5356903 27682880

[118] Hernangómez M Carrillo-Salinas FJ Mecha M Correa F Mestre L Loría F. Brain Innate Immunity in the Regulation of Neuroinflammation: Therapeutic Strategies by Modulating CD200-CD200R Interaction Involve the Cannabinoid System. Curr Pharm Des (2014) 20:4707–22. doi: 10.2174/1381612820666140130202911 PMC4157566 24588829

[119] Amantea D Micieli G Tassorelli C Cuartero MI Ballesteros I Certo M. Rational Modulation of the Innate Immune System for Neuroprotection in Ischemic Stroke. Front Neurosci (2015) 9:147. doi: 10.3389/fnins.2015.00147 25972779 PMC4413676

[120] Cao Y Zhang H Lu X Wang J Zhang X Sun S. Overexpression of MicroRNA-9a-5p Ameliorates NLRP1 Inflammasome-Mediated Ischemic Injury in Rats Following Ischemic Stroke. Neuroscience (2020) 444:106–17. doi: 10.1016/j.neuroscience.2020.01.008 31954830

[121] Sun X Song X Zhang L Sun J Wei X Meng L. NLRP2 is Highly Expressed in a Mouse Model of Ischemic Stroke. Biochem Biophys Res Commun (2016) 479:656–62. doi: 10.1016/j.bbrc.2016.09.157 27693696

[122] Shabanzadeh AP D'Onofrio PM Monnier PP Koeberle PD. Targeting Caspase-6 and Caspase-8 to Promote Neuronal Survival Following Ischemic Stroke. Cell Death Dis (2015) 6:e1967. doi: 10.1038/cddis.2015.272 26539914 PMC4670918

[123] Chidambaran V Costandi A D'Mello A. Propofol: A Review of its Role in Pediatric Anesthesia and Sedation. CNS Drugs (2015) 29:543–63. doi: 10.1007/s40263-015-0259-6 PMC4554966 26290263

[124] Yu Y Jian MY Wang YZ Han RQ. Propofol Ameliorates Calpain-Induced Collapsin Response Mediator Protein-2 Proteolysis in Traumatic Brain Injury in Rats. Chin Med J (2015) 128:919–27. doi: 10.4103/0366-6999.154298 PMC4834009 25836613

[125] Marik PE. Propofol: An Immunomodulating Agent. Pharmacotherapy (2005) 25:28S–33S. doi: 10.1592/phco.2005.25.5_Part_2.28S 15899746

[126] Ma Z Li K Chen P Pan J Li X Zhao G. Propofol Attenuates Inflammatory Damage *via* Inhibiting NLRP1-Casp1-Casp6 Signaling in Ischemic Brain Injury. Biol Pharm Bull (2020) 43:1481–9. doi: 10.1248/bpb.b20-00050 32999158

[127] Wang XJ Cao Q Zhang Y Su XD. Activation and Regulation of Caspase-6 and its Role in Neurodegenerative Diseases. Annu Rev Pharmacol Toxicol (2015) 55:553–72. doi: 10.1146/annurev-pharmtox-010814-124414 25340928

[128] Maas AIR Menon DK Adelson PD Andelic N Bell MJ Belli A. Traumatic Brain Injury: Integrated Approaches to Improve Prevention, Clinical Care, and Research. Lancet Neurol (2017) 16:987–1048. doi: 10.1016/S1474-4422(17)30371-X 29122524

[129] Xu X Yin D Ren H Gao W Li F Sun D. Selective NLRP3 Inflammasome Inhibitor Reduces Neuroinflammation and Improves Long-Term Neurological Outcomes in a Murine Model of Traumatic Brain Injury. Neurobiol Dis (2018) 117:15–27. doi: 10.1016/j.nbd.2018.05.016 29859317

[130] Simon DW McGeachy MJ Bayır H Clark RS Loane DJ Kochanek PM. The Far-Reaching Scope of Neuroinflammation After Traumatic Brain Injury. Nat Rev Neurol (2017) 13:171–91. doi: 10.1038/nrneurol.2017.13 PMC5675525 28186177

[131] Lyman M Lloyd DG Ji X Vizcaychipi MP Ma D. Neuroinflammation: The Role and Consequences. Neurosci Res (2014) 79:1–12. doi: 10.1016/j.neures.2013.10.004 24144733

[132] McKee CA Lukens JR. Emerging Roles for the Immune System in Traumatic Brain Injury. Front Immunol (2016) 7:556. doi: 10.3389/fimmu.2016.00556 27994591 PMC5137185

[133] Tomura S de Rivero Vaccari JP Keane RW Bramlett HM Dietrich WD. Effects of Therapeutic Hypothermia on Inflammasome Signaling After Traumatic Brain Injury. J Cereb Blood Flow Metab (2012) 32:1939–47. doi: 10.1038/jcbfm.2012.99 PMC3463887 22781337

[134] Satchell MA Lai Y Kochanek PM Wisniewski SR Fink EL Siedberg NA. Cytochrome C, a Biomarker of Apoptosis, is Increased in Cerebrospinal Fluid From Infants With Inflicted Brain Injury From Child Abuse. J Cereb Blood Flow Metab (2005) 25:919–27. doi: 10.1038/sj.jcbfm.9600088 15744250

[135] Qu Y Franchi L Nunez G Dubyak GR. Nonclassical IL-1 Beta Secretion Stimulated by P2X7 Receptors is Dependent on Inflammasome Activation and Correlated With Exosome Release in Murine Macrophages. J Immunol (Baltimore Md 1950) (2007) 179:1913–25. doi: 10.4049/jimmunol.179.3.1913 17641058

[136] Wang YC Li WZ Wu Y Yin YY Dong LY Chen ZW. Acid-Sensing Ion Channel 1a Contributes to the Effect of Extracellular Acidosis on NLRP1 Inflammasome Activation in Cortical Neurons. J Neuroinflamm (2015) 12:246. doi: 10.1186/s12974-015-0465-7 PMC4696203 26715049

[137] Speer CP. Pulmonary Inflammation and Bronchopulmonary Dysplasia. J Perinatol (2006) 26:S57–62; discussion S63-4. doi: 10.1038/sj.jp.7211476 16625227

[138] Trittmann JK Nelin LD Klebanoff MA. Bronchopulmonary Dysplasia and Neurodevelopmental Outcome in Extremely Preterm Neonates. Eur J Pediatr (2013) 172:1173–80. doi: 10.1007/s00431-013-2016-5 PMC3742432 23644648

[139] Volpe JJ. Brain Injury in Premature Infants: A Complex Amalgam of Destructive and Developmental Disturbances. Lancet Neurol (2009) 8:110–24. doi: 10.1016/S1474-4422(08)70294-1 PMC2707149 19081519

[140] Felderhoff-Mueser U Bittigau P Sifringer M Jarosz B Korobowicz E Mahler L. Oxygen Causes Cell Death in the Developing Brain. Neurobiol Dis (2004) 17:273–82. doi: 10.1016/j.nbd.2004.07.019 15474364

[141] Hummler JK Dapaah-Siakwan F Vaidya R Zambrano R Luo S Chen S. Inhibition of Rac1 Signaling Downregulates Inflammasome Activation and Attenuates Lung Injury in Neonatal Rats Exposed to Hyperoxia. Neonatology (2017) 111:280–8. doi: 10.1159/000450918 28013306

[142] Vaidya R Zambrano R Hummler JK Luo S Duncan MR Young K. Recombinant CCN1 Prevents Hyperoxia-Induced Lung Injury in Neonatal Rats. Pediatr Res (2017) 82:863–71. doi: 10.1038/pr.2017.160 PMC5874130 28700567

[143] Felderhoff-Mueser U Sifringer M Polley O Dzietko M Leineweber B Mahler L. Caspase-1-Processed Interleukins in Hyperoxia-Induced Cell Death in the Developing Brain. Ann Neurol (2005) 57:50–9. doi: 10.1002/ana.20322 15622543

[144] Dapaah-Siakwan F Zambrano R Luo S Duncan MR Kerr N Donda K. Caspase-1 Inhibition Attenuates Hyperoxia-Induced Lung and Brain Injury in Neonatal Mice. Am J Respir Cell Mol Biol (2019) 61:341–54. doi: 10.1165/rcmb.2018-0192OC 30897338

[145] Yang J Gao J Han D Li Q Liao C Li J. Hippocampal Changes in Inflammasomes, Apoptosis, and MEMRI After Radiation-Induced Brain Injury in Juvenile Rats. Radiat Oncol (London England) (2020) 15:78. doi: 10.1186/s13014-020-01525-3 PMC7147014 32276638

[146] Wallisch JS Simon DW Bayır H Bell MJ Kochanek PM Clark RSB. Cerebrospinal Fluid NLRP3 is Increased After Severe Traumatic Brain Injury in Infants and Children. Neurocrit Care (2017) 27:44–50. doi: 10.1007/s12028-017-0378-7 28181102 PMC5680164

[147] Al Mamun A Wu Y Monalisa I Jia C Zhou K Munir F. Role of Pyroptosis in Spinal Cord Injury and its Therapeutic Implications. J Adv Res (2021) 28:97–109. doi: 10.1016/j.jare.2020.08.004 33364048 PMC7753222

[148] Sahin Ozkartal C Tuzun E Kucukali CI Ulusoy C Giris M Aricioglu F. Antidepressant-Like Effects of Agmatine and NOS Inhibitors in Chronic Unpredictable Mild Stress Model of Depression in Rats: The Involvement of NLRP Inflammasomes. Brain Res (2019) 1725:146438. doi: 10.1016/j.brainres.2019.146438 31518574

[149] Fann DY Lee SY Manzanero S Chunduri P Sobey CG Arumugam TV. Pathogenesis of Acute Stroke and the Role of Inflammasomes. Ageing Res Rev (2013) 12:941–66. doi: 10.1016/j.arr.2013.09.004 24103368

[150] Mahmoudiasl GR Abbaszadeh HA Rezaei-Tavirani M Abdollahifar MA Khoramgah MS Niknazar S. Nod-Like Receptor Protein 3 and Nod-Like Receptor Protein 1 Inflammasome Activation in the Hippocampal Region of Postmortem Methamphetamine Chronic User. Bratisl Lek Listy (2019) 120:769–76. doi: 10.4149/BLL_2019_129 31663353

[151] Harris PA Duraiswami C Fisher DT Fornwald J Hoffman SJ Hofmann G. High Throughput Screening Identifies ATP-Competitive Inhibitors of the NLRP1 Inflammasome. Bioorg Med Chem Lett (2015) 25:2739–43. doi: 10.1016/j.bmcl.2015.05.032 26022841

